# Genomic trade-offs: are autism and schizophrenia the steep price of the human brain?

**DOI:** 10.1007/s00439-017-1865-9

**Published:** 2018-01-15

**Authors:** J. M. Sikela, V. B. Searles Quick

**Affiliations:** 1 University of Colorado School of Medicine, Aurora, CO, USA; 2 Department of Psychiatry, University of California, San Francisco, California, USA

## Abstract

Evolution often deals in genomic trade-offs: changes in the genome that are beneficial overall persist even though they also produce disease in a subset of individuals. Here, we explore the possibility that such trade-offs have occurred as part of the evolution of the human brain. Specifically, we provide support for the possibility that the same key genes that have been major contributors to the rapid evolutionary expansion of the human brain and its exceptional cognitive capacity also, in different combinations, are significant contributors to autism and schizophrenia. Furthermore, the model proposes that one of the primary genes behind this trade-off may not technically be “a gene” or “genes” but rather are the highly duplicated sequences that encode the Olduvai protein domain family (formerly called DUF1220). This is not an entirely new idea. Others have proposed that the same genes involved in schizophrenia were also critical to the rapid expansion of the human brain, a view that has been expressed as “the same ‘genes’ that drive us mad have made us human”. What is new is that a “gene”, or more precisely a protein domain family, has been found that may satisfy these requirements.

## Introduction

Evolution is opportunistic but also indifferent. Changes that become incorporated in a species’ genome need not be without detriment so long as they provide an overall benefit. A consequence of this is that evolution often deals in genomic trade-offs, where harmful effects in some individuals are outweighed by a greater advantage to others. As a result, human disease is sometimes an unfortunate by-product of evolutionary adaptations that have remodeled the genome to incorporate advantageous genomic changes at the expense of introducing disease-causing changes. The classic example of such a genomic trade-off is sickle cell disease and malaria resistance (Allison 1954). A hemoglobin genetic variant, when heterozygous, increases resistance to malaria in populations where the disease is endemic, providing a clear survival advantage. However, this same variant, when homozygous, produces sickle cell disease. Due to its protective effect against malaria, the variant persists in the population, despite its clearly deleterious effects in a subset of individuals.

This concept of genomic trade-offs may similarly apply to the brain and cognitive processes. Such a trade-off might resemble the following: the evolutionary benefits that have resulted from the enhanced cognitive capacity conferred by the human brain have been produced by genomic variants that themselves also give rise to cognitive disorders. This is not a new idea. Over two decades ago, Crow proposed that schizophrenia was a by-product of the key evolutionary events that produced the human brain and language, and that a major effect gene was involved (Crow 1995a, b, 1997, 2000). More recently, Burns has suggested that schizophrenia is an unfortunate by-product of social brain evolution (Burns 2004, 2006, 2007). Both share the view that this serious mental disorder constitutes a costly price paid by our species for our cognitive uniqueness. Their rationale can be expressed as follows: schizophrenia is a highly heritable neuropsychiatric disorder that, while maladaptive, nevertheless persists at high frequency (~ 1%) across virtually all human populations. However, if the disease is genetic and reduces fecundity, why have the underlying genes not been weeded out? This question has been called the central paradox of schizophrenia (Huxley et al. 1964), and Crow and Burns suggest a possible explanation: the causal disease genes also are highly evolutionarily adaptive and, as a result, the disease-causing sequences have been retained in the genome. They further suggest that, because of the high worldwide incidence, it is likely that the key genomic changes occurred prior to the migration of human populations out of Africa and their dispersal across the world. Thus, the crucial genomic variation would be ancient and shared among essentially all human populations. From these observations, they conclude that the key evolutionary benefit of these sequences is that they were critical to the evolution of the human brain.

An excerpt from Burns that quotes an impassioned passage from a novel (Faulks 2005) set in the late 1800s, amid the early days of psychiatry, conveys this rationale:

“The argument for natural selection has long been won, and its basic tenet is that only that which confers an advantage on the species is continually selected by the environment and therefore…perpetuated in the ‘genes’. Nature never selects against the benefit of the species; it cannot. Furthermore, these poor schizophrenic people have…losses in reproduction…Yet they survive at a constant level in the population. How can this be? It breaks the first law of Darwin! It can only be that a variant of that inheritance—the same units, but differently combined, so that they do not express themselves as illness— confer huge advantages. So huge that they compensate both for the misery of the illness, against species interests, and the reproductive failure of the afflicted! And what are these advantages? They are superior brain power, language, creative ability. The same ‘genes’ that drive us mad have made us human: in different combinations, I admit, but precisely, and in my view unarguably, the same particles of inheritance.”

While the cognitive trade-off theory has been proposed previously, new genomic data have become available in recent years that are providing additional support for this possibility. Here, we incorporate some of these new insights to make two testable proposals: (1) the same genes that were responsible for the evolution of the human brain are also a significant cause of autism and schizophrenia, and (2) primary genomic sequences behind this cognitive trade-off are those encoding the Olduvai protein domain family (formerly called DUF1220/*NBPF*) (Sikela and van Roy 2017).

## DNA dosage insights linking autism and schizophrenia

One of the most striking events in the fields of autism and schizophrenia research occurred in 2008 when two independent groups published findings which showed that, of copy number variations (CNVs) in the chromosome 1q21 region, duplications were preferentially associated with autism (and macrocephaly), while reciprocal deletions were preferentially associated with schizophrenia (and microcephaly) (Mefford et al. 2008; Brunetti-Pierri et al. 2008). While both types of CNVs are observed in both disorders, this preferential distribution bias has since been confirmed in several other studies (Crespi et al. 2010). Such findings lent independent support to a previously proposed suggestion that some of the phenotypes of autism and schizophrenia could be viewed as diametric opposites (Crespi and Badcock 2008). Together, these results have several important ramifications:

1. The most plausible explanation for this unusual distribution is that there is a sequence (or sequences) within the 1q21 CNVs, the dosage of which contributes to these two disorders in opposite ways: high-dosage producing autism while low-dosage producing schizophrenia. Such a shared genomic location for autism and schizophrenia has also been found for three other genomic regions where deletions are associated with one disorder, while duplications are associated with the other (Crespi et al. 2010). These results suggest that autism and schizophrenia are related disorders and may have a shared underlying genomic etiology that involves opposite changes in the dosage of the same specific genes.
2. That 1q21 deletions and reciprocal duplications are associated with microcephaly and macrocephaly, respectively, implies that there is a sequence (or sequences) within these 1q21 CNVs, the dosage of which influences brain size in a dose-dependent manner (i.e., more copies produce a larger brain, while fewer copies produce a smaller brain). Such a conclusion also fits well with the fact that autism has been associated with increases in brain size/growth and neuron number (Courchesne et al. 2001, 2003, 2011), while schizophrenia has been linked with reduced brain size (Haijma et al. 2013). Such a pattern also implies that the underlying disease genes have a function related to brain size/growth.
3. Furthermore, if the critical gene(s) contained a highly duplicated and highly copy number variable coding sequence: (a) they would have the capacity to confer a broad spectrum of functional allelic variability that, in theory, could account for the wide range of symptom severity in the disorders and (b) they would have been missed in genetic disease studies because of the difficulty in measuring such sequences. Thus, if one were looking for a primary causal genomic sequence in these disease-associated CNVs, among the most likely would be genes, or parts of genes, that are highly duplicated and copy number polymorphic that have been missed by conventional genomic disease gene searches.
4. In addition, if such sequences were highly adaptive, e.g., also found to be important to the evolutionary expansion of the human brain, they would help to explain the central genetic paradox of schizophrenia, discussed above.

Such a paradox can similarly be applied to autism: a genetic disorder that, while maladaptive, nevertheless persists at a high (> 1%) worldwide incidence (Power et al. 2013).

Do such gene sequences exist? We believe they do. In the following sections, we attempt to show that the sequences encoding the Olduvai protein domain family fulfill these criteria and do so better than any other previously examined sequences in the genome.

## The pond is not dry

It has been reported (Crow 2011) that in 1988, when the power of genetic linkage and association strategies was becoming increasingly appreciated, there was great optimism that the genes underlying neuropsychiatric diseases such as schizophrenia were going to be readily found. This enthusiasm was reflected in the remark that “the thing about these techniques is that they have to work…you drain the pond dry and there are the genes” (Crow 2011). Yet, almost 30 years later, major effect genes for schizophrenia, autism, and other complex cognitive disorders and traits (including cognitive aptitude) continue to remain elusive. Indeed, the existence of a major gene for schizophrenia is currently thought to be “a pretty ridiculous idea” (Barron 2016). In addition, while numerous minor effect loci have been found, they explain only a small fraction of the expected genetic contribution associated with these conditions (Flint 2016). This “missing heritability”, despite efforts to dispel such concerns, continues to be missing.

A key persistent assumption behind the approaches that have been used to identify genetic variants underlying neuropsychiatric and neurodevelopmental diseases [GWAS, exome sequencing, and arrayCGH studies of biallelic CNVs (duplications and deletions that gain or lose a single copy)] has been that they survey the entire genome, and therefore, they “drain the pond dry”. However, that view is incorrect. While these approaches are “genome-wide”, they do not survey the entire genome. Even whole-genome sequencing approaches are limited in their ability to accurately assemble and measure changes in complex, highly duplicated genomic regions, and there are sequences in the genome, among them dynamic, high copy number gene coding regions, that are missed by each of these methods. Contrary to the original expectations, the pond is not “dry”. Notably, Olduvai sequences, which are among the most highly duplicated and copy number polymorphic coding regions in the human genome, have been unexamined by previous genomic studies employing these methods. In only a few instances, as discussed later, have these sequences been directly measured in genetic studies of cognitive capacity and disease (Dumas et al. 2012; Davis et al. 2014a, b, 2015; Searles Quick et al. 2015).

## The cognitive genomic trade-off model of Olduvai domains

In an attempt to reconcile how the same gene sequences could underlie cognitive benefit and cognitive disease, we here make the following testable proposal: which, how, where, and when Olduvai copies change determines whether the consequences are beneficial, innocuous, or harmful.

The Olduvai protein domain family is composed of approximately 300 copies in the haploid human genome, each about 1.4 kb in length (Popesco et al. 2006; O’Bleness et al. 2012), and these are divisible into six primary subtypes based on sequence similarity: CON1–3 and HLS1–3. The domains are found on 23 Neuroblastoma Breakpoint Family (*NBPF*) genes (Vandepoele et al. 2005) where they occur as tandemly arranged units that vary from 6 to 60 copies per gene. The majority of these genes, including the ones that encode the greatest number of Olduvai copies, are found in 1q21.1–1q21.2, a 7 Mb region of chr1. There are 13 *NBPF* genes in this region and they are found interspersed among approximately 40 non-*NBPF* genes.

**Fig. 1:**
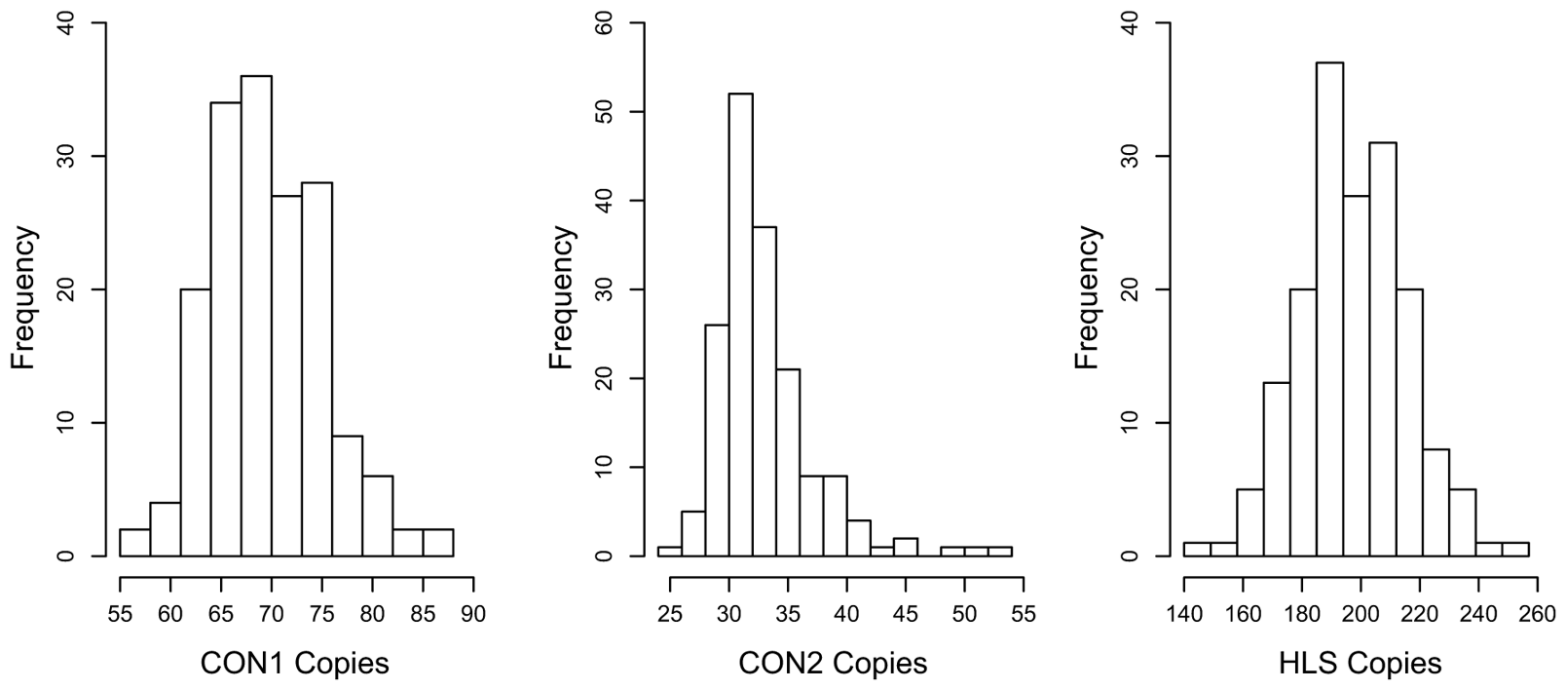
Copy number distribution of Olduvai subtypes in human populations. Bar graph plot showing the diploid copy number distribution for three Olduvai subtypes (CON1, CON2, and HLS1) as determined by ddPCR for 150 unrelated human samples with autism spectrum disorder (Davis et al. 2014b)

**Fig. 2:**
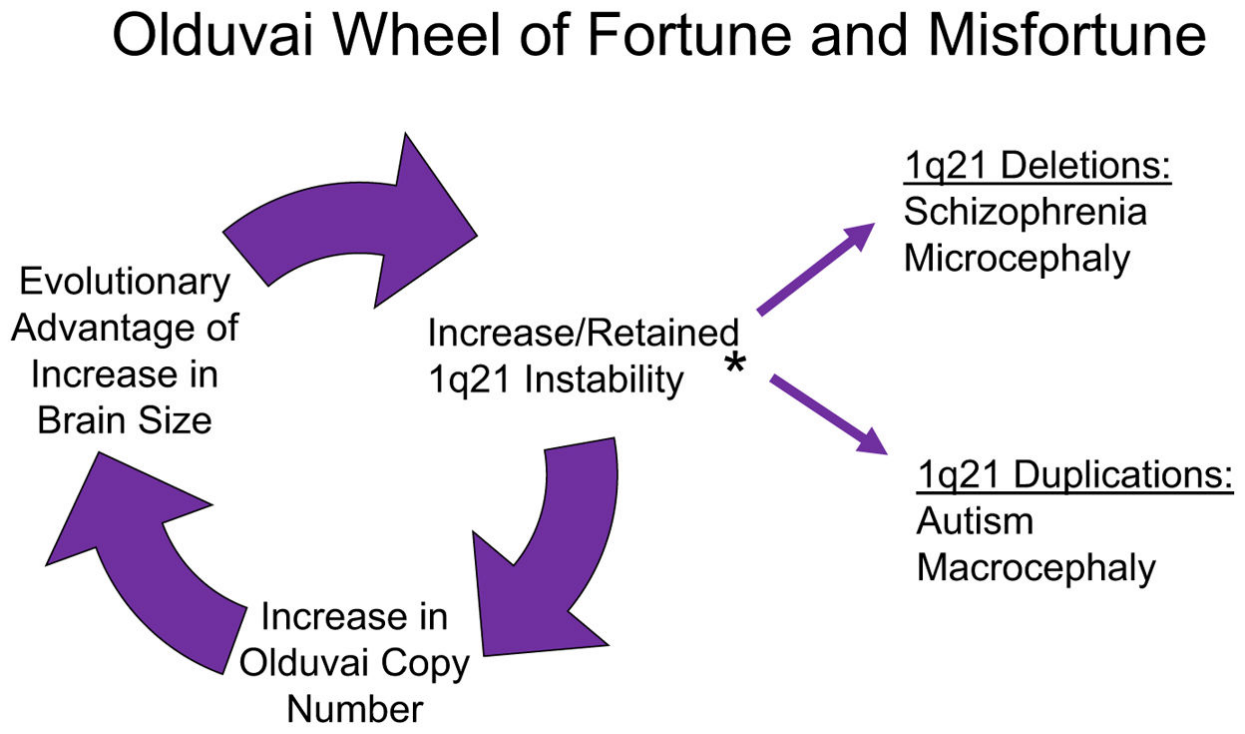
Olduvai wheel of fortune and misfortune. Model depicting how instability of the 1q21 region will increase the chance that beneficial increases in Olduvai copy number will occur. However, when this occurs, it will also result in retention and perpetuation of the unstable region in those who receive the beneficial increase. Such a mechanism will be self-perpetuating, even though it will also be prone to produce detrimental effects in some individuals. The disease-prone nature of the 1q21 region is illustrated by the fact that there are at least 12 different disorders that have been linked to 1q21-associated CNVs *Autism, Congenital Heart Disease, Congenital Anom. of Kidney/Urinary Tract, Epilepsy, Intellectual Disability, Intermittent Explosive Disorder, Macrocephaly, Mayer-Rokitansky-Kuster-Hauser Syn., Microcephaly, Neuroblastoma, Schizophrenia, Thrombocytopenia-absent-radius (TAR) Syn.

Thus, this region contains ~ 250 Olduvai copies that are both tandemly arranged and interspersed among non-Olduvai sequences. Such a genomic architecture would not only be prone to non-allelic homologous recombination (NAHR) events, but there would be a myriad of ways in which the Olduvai/*NBPF* sequences could recombine. Olduvai domains show the largest human lineage-specific increase in copy number of any coding region (approximately 165 copies have been added to the human genome since the *Homo*/*Pan* split) (O’Bleness et al. 2012), and are also highly variable in the human population, exhibiting a broad normal distribution (Fig. 1) (Davis et al. 2014b). These features have multiple important implications with respect to both disease and evolutionary adaptation:

1. Tandemly arrayed sequences are known to be highly variable, with a rate of change that is estimated to be 100,000 faster than that of single-nucleotide substitutions (Ellegren 2000). Small, tandemly arranged coding regions are also known to show accelerated copy number change and have been proposed to contribute to the rapid evolution of morphological traits (Fondon and Garner 2004). Such coding region amplifications and reductions, in contrast to simple single copy deletions or duplications, would have the potential to produce a broad continuum of variation in associated phenotypes.
2. The presence of 13 members of the same gene family (a family composed almost entirely of highly similar tandemly arranged repeated sequences, e.g., Olduvai copies) in close genomic proximity to one another (within 7 Mb) will be prone to multiple aberrant recombination events.
3. Such an architecture can be expected to often produce recombination events that are deleterious, e.g., when two *NBPF* genes undergo NAHR the intervening genes (non-*NBPF*) will either lose or gain a copy as a result. Such gene dosage changes, often involving several genes, will likely produce deleterious phenotypes.
4. If there is a sequence in this region, the increased dosage of which is evolutionary advantageous, such a recombinogenic genomic architecture will increase the probability that a dosage increase of the sequence will occur.
5. If this genomic architecture does lead to an evolutionarily beneficial increase in this sequence (e.g., enhanced survival), the recombinogenic architecture that produced it will be retained in the genome of the individuals who benefitted from the increased copies. In this manner, the beneficial effects will result in retention of the disease-prone genomic architecture. As long as increases in dosage of this sequence continue to be beneficial, such a phenomenon becomes cyclical and lends itself to recurrence. The self-perpetuating nature of this process can be depicted in a model (Fig. 2) (Dumas and Sikela 2009). Indeed, as more tandemly arranged copies are added to the same location (such as occurred with Olduvai sequences in the human genome), the propensity for variation and recombination would increase even further.

**Fig. 3:**
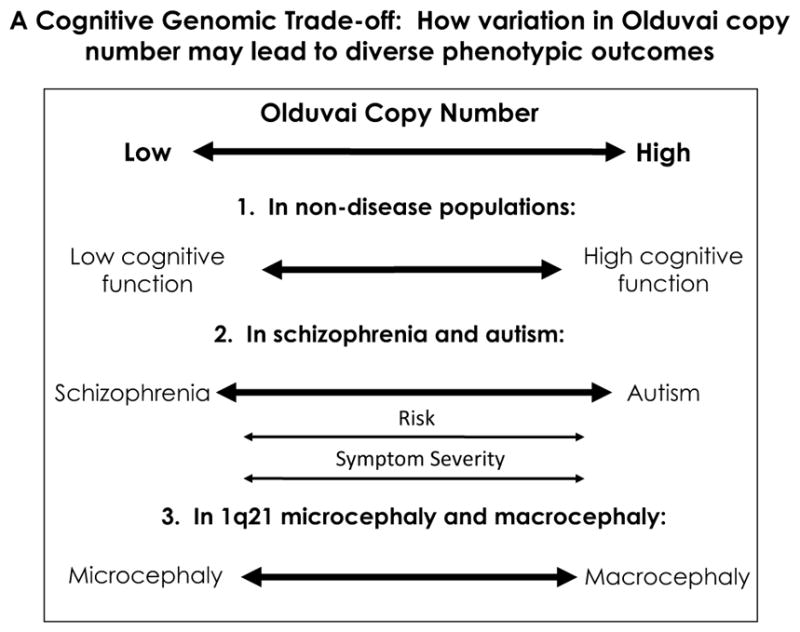
Proposed beneficial and deleterious effects of variation in Olduvai copy number. Shown are the proposed ways in which Olduvai dosage may be contributing in advantageous and detrimental ways to human brain evolution and cognitive disease, respectively. The specific supporting evidence for these relationships is described in separate sections devoted to (1) human brain evolution, (2) autism and schizophrenia, and (3) microcephaly and macrocephaly. The “risk” arrow is meant to show that 1q21-associated duplications and deletions, that encompass many Olduvai copies, have been linked with autism and schizophrenia risk, respectively. The “severity” arrow is meant to show that Olduvai (e.g., CON1) dosage has been linearly associated with the severity of autism and, in the opposite manner with schizophrenia positive symptom severity. While the dosage variation of the Olduvai subtype CON1 is compatible with the proposal that autism symptoms and schizophrenia positive symptoms exhibit diametric phenotypes, a more complete description of the relationships between Olduvai and these disorders is provided in the text

The above factors form the basis of a genomic trade-off model that describes how the beneficial cognitive effects of adding more Olduvai copies can simultaneously produce recurrent genetic diseases. While this possibility was previously suggested (Dumas and Sikela 2009), additional studies have appeared that have lent further support to this model. Incorporating these new findings, the following provides a more detailed explanation for how and why such events may have occurred (Fig. 3). The current model is based on genomic and phenotypic evidence. A key component of future work will be to ascertain the molecular function of Olduvai. There is at present limited evidence pointing to specific cellular functions; what is known will be briefly discussed later in the manuscript.

## Beneficial and harmful effects of Olduvai variation

The following sections outline both the lines of evidence that support Olduvai’s role in evolution and disease, as well as related arguments that should be considered when formulating a model pertaining to genomic trade-offs.

**Fig. 4:**
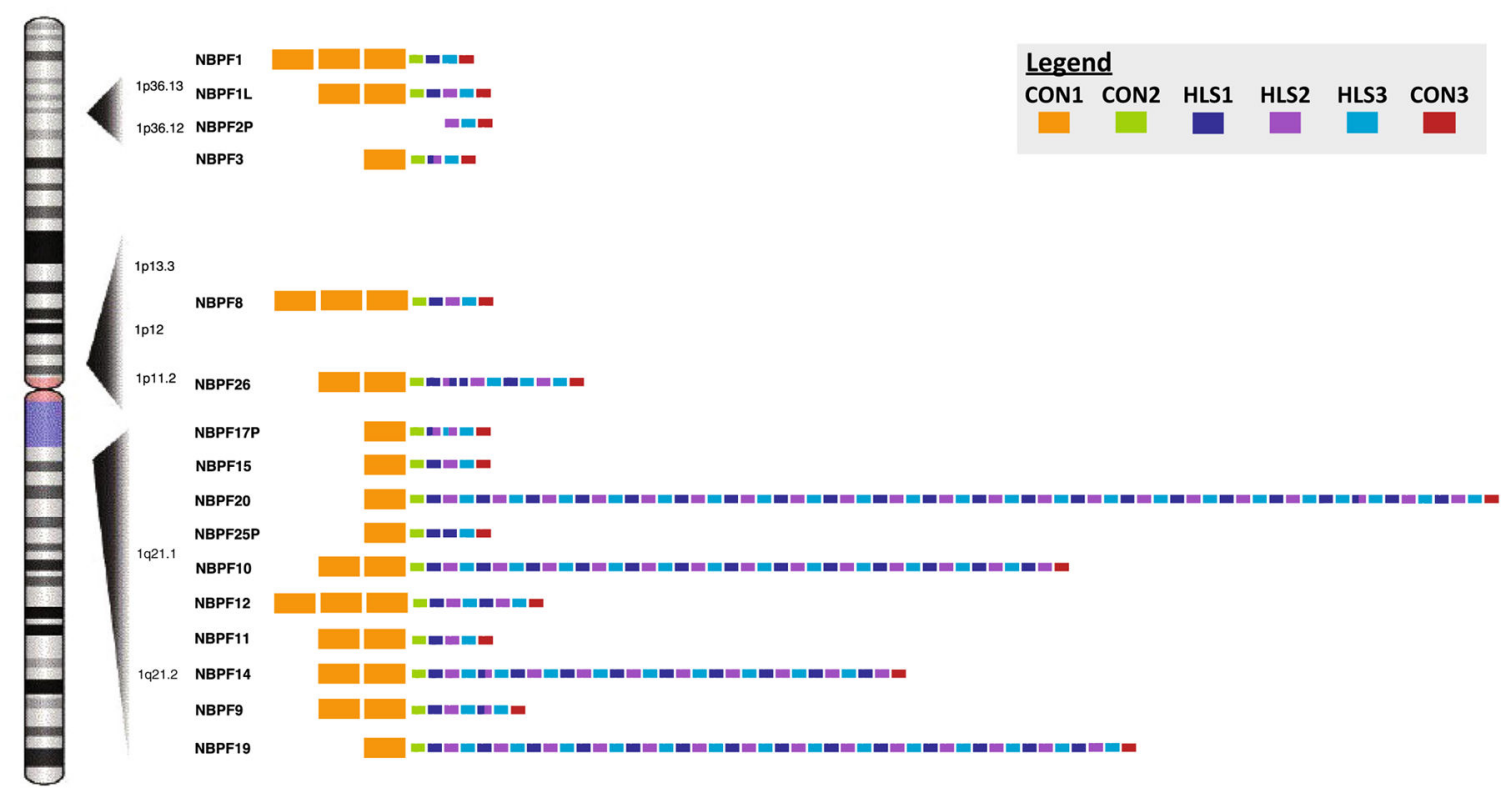
Scattered distribution of CON1 subtypes on chromosome 1. Locations of *NBPF* genes on human chromosome 1 are shown (hg38 assembly) including the organization and number of Olduvai domains (rectangular blocks) predicted to be encoded by each gene. The primary Olduvai subtypes are color-coded, while the block representing the CON1 subtype (yellow) is enlarged

1. Beneficial Olduvai effects: brain evolution and cognitive aptitude
  - As mentioned previously, sequences encoding Olduvai protein domains show the largest human lineage-specific increase in copy number of any coding region in the genome: humans have ~ 300 copies, great apes 90–130, monkeys 30–60, and all other mammals 1–9 (O’Bleness et al. 2012). Olduvai dosage is strongly correlated with an increase in brain size, neuron number, and several other brain size-related phenotypes (but not body size) among primates, and shows a robust linear association with neocortex volume among primate species (O’Bleness et al. 2012; Dumas et al. 2012; Keeney et al. 2014a, b; Zimmer and Montgomery 2015). Such a trend is fully consistent with previous evidence that the evolutionary increase in human brain size represents the extension of a process that began in early primate evolution (Goodman 1999; McKinney 2000) and that the human brain, while remarkable, is essentially a linearly scaled-up primate brain (Herculano-Houzel 2009, 2012a). As Goodman (Goodman 1999) pointed out:
    “Features that we associate with being human did not just arise de novo in the past 6 million years since the lineage to humans separated from that to chimpanzees. Rather, some of the most striking human features, such as greatly enlarged brains and prolonged childhoods in social nurturing societies, have deep roots in our evolutionary history. Forty to 30 million years ago (Ma) neocortical portions of the brain increased in the two emerging branches of anthropoid primates—the platyrrhines (or New World monkeys) and the catarrhines. Within the catarrhine branch, additional marked enlargements occurred by 18–6 Ma in the lineage to the ancestors of modern hominids, and the largest neocortical increases occurred in the past 3 million years in the lineage to modern humans.”
    In addition, there is increasing evidence that primate brain evolution has taken a unique mechanistic path: as the brain expanded from monkeys to apes to humans, neuron number increased, while, unlike in non-primate mammals, neuron size was kept constant (Herculano-Houzel et al. 2007; Herculano-Houzel 2009, 2012b). This primate-specific mode of brain expansion fits well with the fact that there was a burst of Olduvai copy number exclusively in the primate order: anthropoid primates (monkeys, apes, and humans) have 30–300 copies, while non-primate mammals have only 1–9 (O’Bleness et al. 2012).
  - Given that brain size among non-human primate species is also thought to be a predictor of cognitive ability (Deaner et al. 2007), it follows that Olduvai copy number also parallels primate cognitive capacity. As stated by McKinney, “Many of our mental abilities are largely attributable to extension of brain development to produce a proportionately scaled-up version of the ancestral ape brain” (McKinney 2000).
  - Within healthy human populations, increased Olduvai copy number is positively associated with increases in several brain size-related phenotypes (Dumas et al. 2012).
  - Olduvai copy number (subtype CON2) shows a linear association with measures of cognitive aptitude in two independent populations (Davis et al. 2014a, b). While the sample sizes employed in this study were modest, the effect sizes were unusually large. A similar association was also found with a different Olduvai subtype (HLS1) in a separate study (Davis et al. 2015).
  - Other genome-wide studies of cognitive aptitude have had minimal success, finding only minor effect loci that together account for only a small fraction of the expected genetic contribution (Rietveld et al. 2013, 2014; Callaway 2014; Sniekers et al. 2017). None of these studies directly tested the involvement of Olduvai copy number.
  - From an evolutionary perspective, Olduvai protein coding sequences are under strong positive selection, especially among primates (Popesco et al. 2006).
  - Autism has been associated with high intellectual function (Crespi 2016), and it has been reported that the incidence of child prodigy is increased in families in which autism occurs (Ruthsatz and Urbach 2012). To follow up on this, a genome-wide linkage study was undertaken to identify loci that were associated with both autism and prodigy (Ruthsatz et al. 2015). The strongest signal that was obtained was to a region on chr1 where the great majority of Olduvai copies map. While highly repeated sequences are typically invisible to conventional SNP-based analyses (Brahmachary et al. 2014), it is possible that the particular families used in this study contained informative SNPs that allowed these association to be detected.
2. Detrimental Olduvai effects: autism and schizophrenia
  - Olduvai copy number (CON1 subtype) shows a significant positive linear association with increasing severity of a primary symptom of autism, i.e., social impairment, and a suggestive association with diminished communicative skill (Davis et al. 2014b). These findings were subsequently replicated in an independent population (Davis et al. 2015). Thus, as Olduvai copy number increases, the severity of two of the primary symptoms of autism becomes progressively worse. These studies point to the possibility that Olduvai sequences influence autism severity and are doing so in a dosage-related manner (Fig. 3).
  - There is suggestive evidence that supports the possibility that the Olduvai effect size may be large in both autism and schizophrenia. This is based in part on the following observations. The method that was used to link Olduvai copy number with autism and schizophrenia severity (i.e., ddPCR) was limited in two ways: (1) it measured only one Olduvai subtype at a time (e.g., CON1), and (2) it measured only the global (total genome) copy number of the subtype (Davis et al. 2014b, 2015; Searles Quick et al. 2015). CON1 copies are found in almost all *NBPF* genes and these are dispersed over approximately 20 different genomic locations (Fig. 4). As a result, one would be following 20 different loci in each CON1 ddPCR experiment. What is remarkable is that, even with such a broadly based assay, modest sample sizes, and a highly complex phenotype, the positive associations between Olduvai copy number and autism symptom severity were robust enough to be detectable (Davis et al. 2014b) and successfully replicated (Davis et al. 2015). A similar rationale could be applied to the ddPCR studies that linked Olduvai copy number with schizophrenia severity and risk (Searles Quick et al. 2015), and with cognitive aptitude (Davis et al. 2014a). Thus, the fact that such associations are found even using a low-resolution assay, that much of the predicted heritability for these conditions remains unexplained, and that Olduvai sequences have not been directly tested in conventional genomic studies, raises the possibility that they may be major contributors to all three conditions.
  - Studies of Olduvai dosage variation support the view that autism and schizophrenia may be related disorders that have some opposing phenotypic characteristics (Fig. 3) (Crespi et al. 2009). In 1980, Crow suggested that schizophrenia could effectively be separated into positive and negative symptoms (Crow 1980). While the former included hallucinations, delusions and disorganized speech, the latter included flattened affect, poverty of speech, and anhedonia. As mentioned previously, Olduvai studies have found that CON1 copy number is linearly associated with autism severity (Davis et al. 2014b, 2015). Interestingly, in males with schizophrenia, increase in CON1 is also linearly associated with increase in the severity of negative symptoms, which overlap with the symptoms of autism. Conversely, CON1 decreases were associated with increased severity of positive symptoms in individuals with schizophrenia (Searles Quick et al. 2015), as were decreases in another subtype of Olduvai, HLS1. These findings support the aforementioned model that autism and schizophrenia may involve phenotypic extremes that are affected in opposite ways by dosage variation in the underlying genes, and that Olduvai dosage variation may play a role in influencing the severity of both disorders.
  - As mentioned above, CNVs that contain Olduvai sequences have frequently been linked to autism and schizophrenia. However, since 1q21 CNVs have been found in only a small fraction of cases, why would one suggest that Olduvai domains may be a major effect locus? We believe part of the answer is that the methods that have been used to detect genetic variation, including CNVs, would have missed which, how, and where Olduvai copies have changed in the genome. While array-CGH studies that have been reported uncovered associations with autism and schizophrenia, they could only detect changes that involved relatively large genomic segments and were simple in nature (deletions or duplications of one or two copies). They would not be able to follow smaller, multiallelic variations such as those known to occur with the Olduvai family. As a result, these kinds of variations remain unexamined in conventional studies of autism, schizophrenia, and cognitive aptitude.
  - Genomic sequences can range from highly stable to highly variable. Interestingly, it has been shown that there may be a window of variability between these extremes in which sequences can be stable enough to exhibit an obvious heritability yet vary enough that they cannot be reliably followed by SNP-based approaches or conventional linkage and association methods (Brahmachary et al. 2014). Olduvai sequences, which are known to vary considerably in copy number in humans, are excellent candidates to exhibit such behavior. In addition, their instability and capacity to rapidly change would also be consistent with the non-Mendelian nature of these disorders and that the diseases are sometimes discordant in monozygotic twins (Rosenberg et al. 2009; Ahn et al. 2014; Sandin et al. 2014; Colvert et al. 2015). In this regard, it is not surprising that Olduvai sequences have been shown to demonstrate a degree of somatic mosaicism and de novo variation.
  - Finally, the fact that conventional genetic studies of all three conditions (autism, schizophrenia, and cognitive aptitude) have only uncovered minor effect genes/loci that fall well short of accounting for the predicted genetic contribution is consistent with the notion that there are additional causal genes that have not yet been identified. The fact that Olduvai copies have not been examined in virtually all studies of autism, schizophrenia, and cognitive aptitude, together with the other features discussed above, makes them compelling candidates to explain a portion of the missing heritability associated with each condition.
  - When considering the detrimental effects of Olduvai in discussions of autism and schizophrenia, an obvious question is why no clear difference has been observed in global copy number between individuals with autism and controls (Davis et al. 2014b). This may also be explained by the nature of the ddPCR assay. Because the method only provides global copy number for CON1, it does not address which specific CON1 copies are changing, nor where in the genome such changes are occurring (e.g., in which specific *NBPF* genes) or whether variations are due to germline or somatic copy number changes. If such factors are critical to whether the effects produce disease or not, they will not be detected using ddPCR or similar methods that measure only global Olduvai copy number. And as discussed above, conventional assay techniques used in prior studies of these disorders have not examined Olduvai copy number variation.
3. Detrimental Olduvai effects: microcephaly and macrocephaly
  - It was previously mentioned that multiple reports have linked 1q21-associated duplications and reciprocal deletions to macrocephaly and microcephaly, respectively (Mefford et al. 2008; Brunetti-Pierri et al. 2008). While these CNVs contained a substantial number of genes, it was later noted that they also contained many Olduvai copies (Dumas and Sikela 2009).
  - Subsequently, a comprehensive analysis determined that, of the 50 genes located in the 1q21 region, Olduvai copy number showed the strongest association with brain size in individuals with 1q21-associated microcephaly and macrocephaly (Dumas et al. 2012). This finding was the first to demonstrate a clear deleterious effect to Olduvai copy number variation.
  - In an apparent contradiction to this trend, an individual was recently identified with a large (8.3 Mb) 1q21.1–1q21.3 duplication and microcephaly (Milone et al. 2016). However, the individual also had a 50 kb microdeletion embedded within the duplicated region which contained only two genes, both of which encode Olduvai domains: *NBPF25P* and *NBPF23*. Thus, in this case of microcephaly, which involved a duplicated segment encompassing more than 120 genes, the only genes that were deleted encoded Olduvai copies.
  - In reports of 1q21 duplications and deletions, the detected CNVs encompass numerous non-*NBPF* genes in addition to several *NBPF* genes that encode Olduvai domains. Thus, while Olduvai gains and losses may underlie changes in brain size in 1q21-associated macro/microcephaly, it is likely that the dosage change of the flanking (i.e., non-*NBPF*) genes is responsible for the diverse pathologies that have been found among these individuals.
  - The recombinogenic architecture of the 1q21 region that results from the many Olduvai copies that map there would be expected to produce many deleterious recombination events. This expectation is borne out by the reports of at least 12 different disorders that have been associated with 1q21 CNVs (Dumas et al. 2012).

Given the above considerations, our primary proposal to explain how Olduvai domains could be a key factor in both beneficial (cognitive capacity) and harmful (autism and schizophrenia) effects is as follows: which, where, how, and when Olduvai copies are changing determines whether beneficial, neutral, or harmful effects will occur. Such a model is not without precedent. The red and green opsin genes are also found tandemly arranged in the human genome and the high frequency of recombination between them is responsible for many peculiarities of red–green color vision in humans including the extraordinarily high frequency of color vision defects (Neitz and Neitz 2011).

## Are there other genes that fit the cognitive trade-off model?

The overwhelming trend emerging from genetic studies of autism, schizophrenia, and cognitive aptitude has been that each of these conditions involves hundreds-to-thousands of small effect genes and/or loci. However, even using very large sample sizes, the identified loci fall far short of fully accounting for the expected genetic contribution to these conditions. For example, in the largest study of cognitive aptitude, involving over 76,000 individuals, 52 loci were identified, but they account for less than 5% of the expected genetic contribution to this phenotype (Sniekers et al. 2017). Similar results have been found for autism (Yuen et al. 2017) and schizophrenia (Crow 2011; Flint 2016). However, as mentioned previously, these conventional genome-wide studies do not look at the entire genome and none have directly measured Olduvai sequences.

One of the few examples of another gene that potentially fits the cognitive genomic trade-off model is the *ARHGAP11B* gene (Florio et al. 2015). While human-specific sequence variation in the gene has been implicated in promoting neuron increases, the gene is also found in a genomic location, 15q13, that has long been thought to harbor sequences that contribute to schizophrenia (Stefansson et al. 2008). However, in this example, human brain enlargement is proposed to be due to a single base change (Florio et al. 2016). Such a small change does not offer the genomic variability that would be needed to explain the continued, gradual, prolonged nature of primate brain expansion and the spectrum of symptom severity associated with schizophrenia (not to mention the range of severity associated with autism and the variation associated with cognitive aptitude…data that, in contrast, fits well with the extreme copy number variability of Olduvai in humans).

As mentioned, the possibility that there may be a major effect gene involved in schizophrenia has been proposed previously (Crow 1995b), and is in part based on Karlsson’s analysis of the incidence of schizophrenia in multigenerational Icelandic populations (Karlsson 1974, 1982, 1988). He states, “The segregation observed in certain large kindreds into branches with high and low rates of psychosis is very suggestive of a modified dominant transmission. The gene frequencies that would have to be postulated for modified recessive or polygenic inheritance would not permit such a pattern. A system based on a dominant principal gene, perhaps associated with other modifying genes, thus seems to be the most likely mode of transmission.”

While there were many fruitless searches for such a major gene, the lack of success may have been not because a major gene did not exist, but rather because the early genomic surveys that were carried out used methods that were very limited in scope. What were missed, then, and continue to be unexamined in current conventional genomic searches, are highly duplicated and dynamic sequences such as those that encode the Olduvai family.

The proposal that Olduvai sequences may represent a major effect locus for these conditions is not meant to imply that other genetic loci are not also involved. It is possible that other major effect loci have been missed, due to the same limitations that previously hindered the discovery of Olduvai. In addition, just as with any complex phenotype, normal brain function requires the action of multiple molecular components, any one of which, when faulty, will result in a sub-optimal outcome. Thus, if Olduvai variation is a primary contributor to these conditions, it is quite plausible that the many minor effect genomic variations that have been found may involve loci that affect the same pathways, perhaps upstream or downstream of Olduvai action. However, such loci will be scattered across the genome and, therefore, sorted independently. As a result, their contributions may only account for a small fraction of the expected genetic contribution to these conditions. A similar argument can be made with respect to the genetics of autism and cognitive aptitude.

Finally, it is worth noting that: (1) none of the identified genes or loci implicated in schizophrenia, autism, and cognitive aptitude show as great a capacity for functionally important allelic variation as the Olduvai family; (2) none show as great a human lineage-specific increase in copy number; and (3) none show a stronger correlation with the progressive, continual increase in brain size that is found during the evolution of the primate order and that has reached its most extreme form in the human lineage.

## What key issues remain?

There are two primary needs related to Olduvai domains that should be addressed:

### Understand Olduvai function

From a functional perspective, little is definitively known regarding the cellular role of Olduvai. While some functional clues for these sequences have emerged (Popesco et al. 2006; Dumas et al. 2012; Zhu et al. 2017), their precise role in human brain function remains unclear. An early human study found that Olduvai domains are expressed in several tissues including at high levels in brain, where they show neuron-specific expression (for details, see Popesco et al. 2006). Previous work examining the cellular function and expression timing of Olduvai has suggested a role in promoting neuronal stem cell proliferation (Keeney et al. 2014a), findings that are quite compatible with the strong correlative data linking Olduvai dosage with brain size. A recent study similarly found that *NBPF7* upregulation promotes cellular proliferation (Zhu et al. 2017). Interestingly, the majority of identified microcephaly disease genes encode centrosomal proteins (Bond and Woods 2006), and several lines of evidence have linked Olduvai function to the centrosome (Dumas et al. 2012). Prior studies have also attempted to elucidate *NBPF* and Olduvai function using techniques such as co-immunoprecipitation, transgenic mouse models, and *in silico* sequence analyses (Vandepoele et al. 2010; Keeney et al. 2015; Zhou et al. 2013), but few results have been replicated in the current literature.

Given that the domain family in humans has 300 haploid copies that are distributed among 23 dispersed human genes, it is not surprising that the sequences have been challenging to study at the functional level. However, we strongly discourage the use of genetic strategies that involve “humanizing” nonhuman primates as a means of understanding Olduvai function (Coors et al. 2010). Much new knowledge can be obtained without using living animal models. Progress in answering such questions may be aided by the availability of human iPS cells and the ability to precisely modify their genome.

### Develop better genomic technologies for studying and measuring Olduvai domains and other highly duplicated sequences

There are two reasons why this would be beneficial. First, no human or primate genome sequence assembly is complete. Regions that are highly duplicated are often not accurately assembled. Second, what is being missed is important. Some of the sequences that are being missed are likely to be key contributors to human disease and human evolution. Our ability to predict whether Olduvai changes are beneficial or harmful may depend on our ability to determine the precise nature of Olduvai variation in the genome.

## The costly price of the human brain

Here, we provide support for a model that the Olduvai protein domain family may underlie a cognitive genomic tradeoff in our species. Specifically, we propose that Olduvai sequences can play both a beneficial role (in brain evolution and cognition) and a detrimental role (in autism and schizophrenia), and which outcome occurs depends on which, where, how, and when copies are changing. This duality of effect can potentially account for the central paradox of why autism and schizophrenia, two genetic but maladaptive disorders, persist at high frequency across human populations. In addition, increases and decreases in Olduvai (e.g., CON1 subtype) copy number mirror the partially opposing phenotypic profiles that are associated with autism and schizophrenia. Finally, the high copy number and dynamic, polymorphic nature of Olduvai coding sequences makes them a rich source of unexamined functional allelic variation. As such, they represent an excellent candidate to explain why genome-wide studies of autism, schizophrenia, and cognitive capacity have not accounted for the predicted genetic contribution of these conditions.

Should this model be proven true, and our understanding of autism and schizophrenia requires that we study genes also involved in human brain evolution and cognitive capacity, we owe it to those afflicted with these disorders to do so. They, through no choice of their own, must carry these burdens. It may well be the steep price evolution has placed on the human brain, and we should feel compelled to show a greater compassion and support for those who have paid, and continue to pay, for its existence.
